# Prognostic Significance of Chronic Kidney Disease (CKD-EPI Equation) and Anemia in Patients with Chronic Heart Failure Secondary to Chagas Cardiomyopathy

**DOI:** 10.1155/2020/6417874

**Published:** 2020-07-06

**Authors:** Marcelo Arruda Nakazone, Maurício Nassau Machado, Ana Paula Otaviano, Ana Maria Silveira Rodrigues, Augusto Cardinalli-Neto, Reinaldo Bulgarelli Bestetti

**Affiliations:** ^1^Postgraduate Division, São José do Rio Preto Medical School, 5416 Brigadeiro Faria Lima Ave., CEP 15090-000, São José do Rio Preto, Brazil; ^2^Hospital de Base, Fundação Faculdade Regional de Medicina de São José do Rio Preto, 5544 Brigadeiro Faria Lima Ave., CEP 15090-000, São José do Rio Preto, Brazil; ^3^Hospital das Clínicas, Ribeirão Preto Medical School, University of São Paulo, Campus Universitário, Ribeirão Preto CEP 14048-900, Brazil; ^4^Specialized Nursing Department, São José do Rio Preto Medical School, 5416 Brigadeiro Faria Lima Ave., CEP 15090-000, São José do Rio Preto, Brazil; ^5^University of Ribeirão Preto, 2201 Costábile Romano Ave., CEP 14096-900, Ribeirão Preto, Brazil

## Abstract

**Background:**

Few studies regarding chronic kidney disease (CKD) and anemia have been conducted in patients with Chagas cardiomyopathy (CC). We evaluated the risk prediction performance of the Chronic Kidney Disease Epidemiology Collaboration (CKD-EPI) equation and anemia in CC patients.

**Methods:**

From 2000 to 2010, a total of 232 patients were studied in a single-center retrospective study. CKD was defined as creatinine clearance <60 mL/min/1.73 m^2^ according to CKD-EPI equation. Anemia was defined as hemoglobin <12 g/dL (women) and <13 g/dL (men). Cox proportional hazards models were used to establish predictors for death.

**Results:**

At baseline, 98 individuals (42.2%) had criteria for CKD and 41 (17.7%) for anemia. During follow-up, 136 patients (58.6%) died. Independently, CKD and anemia were not associated with all-cause mortality. However, when they coexisted, an additional risk was attributed for these patients. Cox proportional hazard models analysis identified systolic blood pressure (hazard ratio, 0.99; 95% confidence interval (CI), 0.98 to 1.00; *P*=0.015), implantable cardioverter-defibrillator (hazard ratio, 0.48; 95% CI, 0.27 to 0.85; *P*=0.012), left anterior fascicular block (hazard ratio, 1.52; 95% CI, 1.08 to 2.13; *P*=0.017), left ventricular end-diastolic diameter (hazard ratio, 1.04; 95% CI, 1.02 to 1.06; *P* < 0.001), and serum sodium (hazard ratio, 0.95; 95% CI, 0.92 to 0.99; *P*=0.020) as independent predictors for death.

**Conclusions:**

CKD and anemia are not independent predictors for long-term mortality in CC patients. However, the prognosis is poorer in individuals with both comorbidities.

## 1. Introduction

Chronic systolic heart failure (CHF) is an insidious syndrome that results in a varying degree of functional impairment, despite the modern CHF therapy [1]. CHF secondary to Chagas cardiomyopathy (CC) has a poor prognosis compared to other etiologies [2, 3], and unfortunately CC remains the leading cause of CHF in areas where the disease is endemic [4].

The chronic kidney disease (CKD) has been associated with significantly increased risks of cardiovascular disease morbidity and mortality, even at its earliest stage [5]. Glomerular filtration rate (GFR) is the best overall index used in the diagnosis, evaluation, and management of CKD [6]. In 2009, a new estimating equation for GFR was proposed by the Chronic Kidney Disease Epidemiology Collaboration (CKD-EPI) [7], providing better risk predictions.

Anemia is a common comorbidity in CHF patients and is associated with poorer prognosis [8]. Al-Ahmad et al. [9] found that CKD and anemia are independent risk factors for death among patients with CHF enrolled in the SOLVD clinical trial, in disagreement with previous studies that did not observe contribution of anemia to the risk of death [10, 11]. The CHF commonly causes renal impairment and, to this vicious circle is often added anemia, which can be produced not only by CKD but also by CHF, thus worsening both conditions [12].

In this context, we aimed to evaluate the long-term mortality risk stratification performance of the CKD-EPI equation and anemia in patients with CC and to determine the independent predictors of all-cause mortality in our population.

## 2. Materials and Methods

### 2.1. Patients Selection

This single-center study retrospectively evaluated patients with two positive serologic tests for Chagas disease (ELISA and indirect immunofluorescence), according to the recommendation of the World Health Organization [13]. The diagnosis of CHF has been made by the attending physicians using the Framingham Criteria for Heart Failure Diagnosis [14]. After clinical diagnosis of CHF, a 2D echocardiography was performed in each patient to confirm the clinical diagnosis, quantify this condition using left ventricular ejection fraction (LVEF), and guide the treatment. Individuals with clinical diagnosis for CHF secondary to CC and LVEF <55% on 2D echocardiography confirming left ventricular systolic dysfunction were screened for this study. Patients with a concomitant disease that could potentially cause heart disease by itself were excluded. The methodology of this investigation is consistent with the STROBE checklist for observational studies.

This study was conducted in accordance with the Declaration of Helsinki and approved through the local Human Research Ethics Committee of São José do Rio Preto Medical School (CAAE-02716112.6.0000.5415). The need for individual informed consent was waived, as this study was a retrospective analysis of prospectively collected data for routine care, and breach of privacy or anonymity did not occur.

### 2.2. Baseline Measurements

The demographics data, New York Heart Association (NYHA) functional class, heart rate, systemic arterial pressure, medical history, standard laboratory tests, 12-lead resting electrocardiogram, and cardiac electronic implantable devices information were noted at study entry and were retrieved from medical charts records.

Anemia was defined as hemoglobin <12 g/dL for women and <13 g/dL for men [15]. The creatinine clearance was estimated according to CKD-EPI equation [7] and CKD was defined as a creatinine clearance <60 mL/min/1.73 m^2^.

### 2.3. Prospective Follow-Up

The patients were routinely followed from January, 2000, to December, 2010, at Cardiomyopathy Outpatient Service, Hospital de Base, São José do Rio Preto Medical School, a public referral center for severe CHF management in the northwest of São Paulo, Brazil. The CHF medical therapy information was retrieved from a prospectively collected database of patients. All patients received evidence-based treatment for CHF (angiotensin converting enzyme inhibitors or angiotensin receptor blocks and beta-blockers at targeted or maximal tolerated doses), according to the international guidelines of the time. Patients usually visited the outpatient service each four months, and a senior heart failure expert supervised the treatment given. Patients were censored at heart transplantation or death.

### 2.4. Data Analysis

The data were analyzed using the IBM SPSS Statistical Package v.21 (IBM Corporation, Armonk, NY). The variables are presented as absolute numbers and percentages or median and interquartile ranges (25^th^ and 75^th^ percentile) when applicable. Due to the lack of Gaussian distribution, continuous variables were compared using the nonparametric Mann–Whitney *U* test. Chi-square or Fisher's exact tests were used to compare categorical variables. We did not use any method of data imputation.

Univariate and multivariable Cox proportional hazards models (stepwise backward elimination method) were used to determine independent predictors for all-cause mortality during a long-term follow-up. After univariate analysis, variables with clinical relevance and *P* < 0.10 were included in the multivariate model. Continuous variables underwent the Spearman test to establish correlation among them. The variable which correlated with others and with the highest Wald coefficient remained in the model, whereas the other was ruled out. Thus, each variable entered the multivariable model in a proportional to 10 events in an attempt to avoid overfitting. The multivariate model was then adjusted for age, gender, NYHA functional class, heart rate (beats/minute), systolic and diastolic blood pressures (mmHg), need for implantable cardioverter-defibrillator (ICD), left anterior fascicular block on 12-lead resting electrocardiography, left ventricular end-diastolic diameter (mm), serum sodium level (mEq/L), anemia status, and CKD according to eGFR CKD-EPI. The adjusted hazard ratio (HR) and 95% confidence interval (95% CI) were calculated for the predictors. Cumulative survival graphic (Kaplan–Meier) was constructed to show differences in event-free survival between patients according to the presence of CKD and anemia associated to NYHA functional classes. *P* values <0.05 were considered statistically significant (two-tailed).

## 3. Results

A total of 234 patients were initially screened for the study. Two patients with no hemoglobin measurement were ruled out of the investigation. Thus, 232 individuals (65.9% male) who had a median age of 56 years (45–66) and fulfilled inclusion criteria entered the study. The baseline characteristics of patients are shown in Table 1.

These individuals were divided into groups: CKD and non-CKD, according to eGFR CKD-EPI and anemic and nonanemic, according to hemoglobin serum levels. Ninety-eight patients (42.2%) had renal dysfunction, whereas 41 (17.7%) had anemia. Patients with CKD status were older (median for age = 63 years), had higher right ventricular diameters (median = 27 mm), showed lower spontaneous heart rate (median = 66 beats/min), and, consequently, had more need for pacemaker (63.3%) at start of the outpatient follow-up compared to non-CKD group (median for age = 52 years, *P* < 0.001; median = 23 mm, *P*=0.011; median = 70 beats/min, *P*=0.026; and 44.0%, *P*=0.001, respectively). Anemic patients were older (median for age = 63 years) and had higher rate (12.2%, data not shown in Table) for end-stage renal disease (eGFR CKD-EPI < 30 mL/min/1.73 m^2^) compared to nonanemic individuals (median for age = 55 years, *P*=0.010 and 3.1%, *P*=0.028, respectively). The other laboratory tests, 2D-echocardiographic and 12-lead resting electrocardiography findings observed at study entry were not associated with CKD or anemia status (Table 2).

Clinical complications as hospitalization, cardiogenic shock, and need to heart transplantation were similar between patients with CKD and anemia (*P* > 0.05 for all subgroups). During follow-up (median of 799 days, interquartile range of 291 to 1441 days), 136 patients (58.6%) died. Similar rates for late-mortality were shown by individuals with CKD (60.2%) and anemia (68.3%) compared to non-CKD (57.5%, *P*=0.675) and nonanemic patients (56.5%, *P*=0.166), respectively.

After adjustment, the cox proportional hazard model analysis identified five variables as independent predictors for all-cause mortality: systolic blood pressure (HR = 0.99; 95% CI, 0.98 to 1.00; *P*=0.015), use of implantable cardioverter-defibrillator (HR = 0.48; 95% CI, 0.27 to 0.85; *P*=0.012), left anterior fascicular block (HR = 1.52; 95% CI, 1.08 to 2.13; *P*=0.017), left ventricular end-diastolic diameter (HR = 1.04; 95% CI, 1.02 to 1.06; *P* < 0.001), and serum sodium level (HR = 0.95; 95% CI, 0.92 to 0.99; *P*=0.020) (Table 3). Interestingly, anemia and CKD status were not retained in the multivariate model as independent predictors.

**Figure 1:**
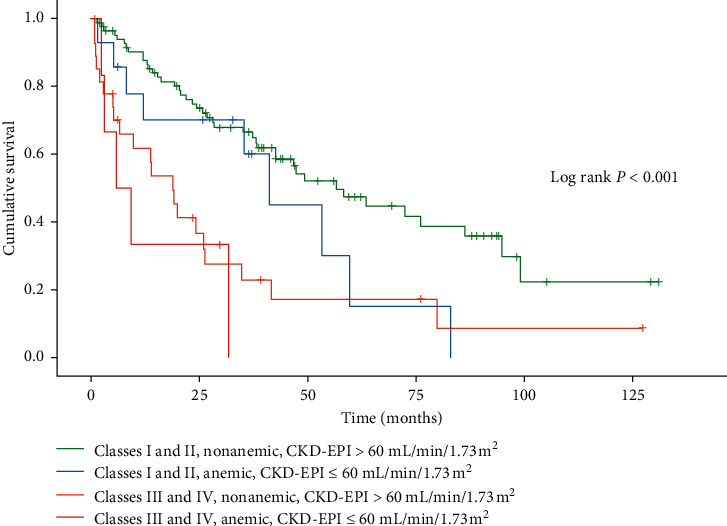
Survival probabilities of patients with Chagas cardiomyopathy according to chronic kidney disease and anemia status associated with New York Heart Association Functional Classes.

Probability of survival for patients with CKD was 73.3%, 58.2%, 49.8%, and 33.6% at 12, 24, 36, and 60 months, respectively, and for non-CKD patients was 83.0%, 67.3%, 56.5%, and 39.5% at 12, 24, 36, and 60 months, respectively (*P*=0.254). The probability of survival for anemic patients was 72.9%, 64.9%, 52.1%, and 29.2% at 12, 24, 36, and 60 months respectively, and for nonanemic patients was 80.3%, 63.1%, 53.9%, and 38.7% at 12, 24, 36, and 60 months, respectively (*P*=0.0111). A lower survival probability for patients with CC according to functional classes of CHF was observed. Moreover, CKD and anemia status significantly showed an additional impact on survival for patients with CC (*P* < 0.001, Figure 1).

## 4. Discussion

In our study, we evaluated the long-term mortality risk stratification performance of CKD and anemia in outpatient individuals with CC. Although previous studies have addressed these variables on the prognosis of patients with CHF secondary to CC, this work is the first cohort of Brazilians assessed using the eGFR CKD-EPI equation that provides more accurate estimates and better predictive power. Our investigation clearly showed that survival probabilities of patients with CHF secondary to CC, allocated into the same group for NYHA functional classes, are lower in those with CKD and anemia, particularly in severe CHF individuals. Nonetheless, neither CKD nor anemia is an independent predictor for all-cause mortality in patients with CC, suggesting the poorer prognosis of this condition.

The CHF secondary to CC is a major public health problem in Latin America, where about 10,000 people die of this disease annually [16], causing a profound socioeconomic impact [17]. CKD is a common comorbidity in CHF patients and is associated with the disease severity, worse prognosis, and higher anemia prevalence [18, 19]. Nevertheless, there are few published studies relating CKD and anemia with CHF in patients with CC. In our series, the prevalence of CKD was 42.3%, similarly to that observed in non-Chagas population enrolled in clinical trials or data obtained from prospective longitudinal cohort studies [20–22].

Compared to non-CKD individuals, those with this condition were older, had higher right ventricular diameters, showed lower median for spontaneous heart rate, and, consequently, had more need for pacemaker, suggesting higher severity of CC. However, as previously reported by our group [23] and Ferreira et al. [24], this investigation did not confirm the isolated association between CKD and worse outcomes, including mortality. The younger status of our patients with no underlying ischemic conditions (coronary artery disease and peripheral and/or cerebrovascular diseases) may account, at least in part, for our different results. Moreover, there was a higher proportion of patients on renin-angiotensin-aldosterone blockade [21] at maximal tolerated doses according to guidelines recommendations and individuals with chronic systolic dysfunction only, managed in specialized heart failure outpatient clinic, facts that may have contributed to the reduction of renal impairment influence on mortality.

In end-stage renal disease population, anemia is a well-recognized risk factor for all-cause mortality [25], occurring mainly due to erythropoietin deficiency. In addition, anemia also occurs in individuals with less severe renal dysfunction [9] in several other disorders, as bone marrow depression, that interfere with the action of erythropoietin and cellular release and utilization of iron [26]. Our investigation showed a prevalence of 17.7% of anemia in CC population, a slightly higher rate compared to similar Brazilian cohort [27]. However, this prevalence may vary from 4 to 69.7%, depending on the diagnostic criteria and the study population, increasing in accordance to age and severity of CHF and other comorbidities, as nutritional status and low weight patients [8, 28]. On the other hand, our data are consistent with those of Miguel et al. [29], who studied a smaller population of CC patients with CHF.

Furthermore, our findings were opposite to results described by Ferreira et al. [24] which evidenced high prevalence of anemic patients with CHF and an isolated significant impact on their survival, even for mild degree of anemia. Although in distinct population, Al-Ahmad et al. [9] hypothesized four potential explanations for poorer prognosis in these individuals, level of hematocrit may be an additional marker of cardiac function, severe CHF may cause anemia through undefined mechanisms, and reduced hematocrit may be a risk factor for ischemia, worsening this manifestation, mainly in organisms with preexisting heart disease and may result in ventricular remodeling and cardiac dysfunction, culminating in a vicious cycle [30].

Anemic patients were older and showed higher rate for end-stage renal disease compared to nonanemic individuals, emphasizing the known association between these comorbidities [25, 31]. Moreover, the subgroup analysis showed that patients allocated into the same group for NYHA functional classes had lower survival probability when CKD and anemia coexist with CHF, evidencing the burden of these conditions. However, in our series, neither CKD nor anemia were independent predictors of worse outcomes, including hospitalizations, cardiogenic shock, need to heart transplantation, and mortality. This finding suggests that they are markers and not independent risk-factors for all-cause mortality in Brazilian patients with CC [29].

In the multivariate model, left ventricular end-diastolic diameter and left anterior fascicular block were positively associated with mortality, confirming previous findings and well-known risk factors [32]. On the other hand, our investigation showed systolic blood pressure, use of ICD, and sodium serum level as independent protective factors for mortality. In accordance with our results, there was one percent of risk reduction at each elevation of 1 mmHg of systolic blood pressure. One possible explanation for the protective effect is the fact that patients with higher blood pressure undergo pharmacologic treatment considering higher doses of renin-angiotensin-aldosterone blockade drugs and beta-blockers, therapy with known survival improvement effect [32, 33]. The fact that CC may be considered as a type of a catecholaminergic cardiomyopathy [34], which is reversed by beta-blockers [35] lends further support to this assumption. Regarding the use of ICD, we believe that the main reason for this finding is the prevention of sudden cardiac death due to life-threatening ventricular arrhythmias, common clinical complication in patients with severe CHF secondary to CC [4, 36]. Furthermore, as previously demonstrated by our group, hyponatremia is an independent predictor of all-cause mortality for this population [37] and may appear as a consequence of marked activation of the renin-angiotensin-aldosterone and autonomic nervous systems, which ultimately determines myocyte death, reparative fibrosis, and ventricular remodeling [38, 39]. In this context, maybe is prudent to avoid hyponatremia to counteract the deleterious effect of activation of the involved systems.

Our study has several limitations. First, the investigation was a retrospective view of a prospective patients' cohort. Therefore, unmeasured factors may have biased our findings. Second, we did not determine the etiology and the incidence of worsening CKD. Detecting worsening renal dysfunction over time would be interesting to detect potential association with death. Finally, we did not investigate the specific causes of anemia, including iron, folate, and vitamin B12 deficiencies, dilutional anemia, and the anemia of not surveyed chronic diseases. On the other hand, the data were prospectively collected, and the statistical analysis performed appears to have avoided the overfitting phenomenon, thus making our date reliable. Besides, our sample size was reasonable, and patients received evidence-based treatment, thus reflecting the contemporary era of CHF treatment.

## 5. Conclusions

In summary, CKD and anemia are not independent predictors for long-term mortality in patients with CHF secondary to CC that, by itself, has a worse prognosis. However, patients with these comorbidities have lower survival probabilities, in spite of their respective NYHA functional classifications.

## Figures and Tables

**Table 1:** Baseline characteristics of Chagas cardiomyopathy prospective cohort (*N* = 232).

Variable	Median (25^th^–75^th^) or *N* (%)
Clinical characteristics	
Age (years)	56 (45–66)
Gender (male)	153 (65.9)
NYHA classes I and II	157 (67.7)
NYHA classes III and IV	75 (32.3)
Heart rate (beats/min)	68 (60–80)
Systolic blood pressure (mmHg)	110 (100–120)
Diastolic blood pressure (mmHg)	70 (60–80)
Type 2 diabetes mellitus	11 (4.7)

Laboratory analysis	
Sodium (mEq/L)	141 (138–144)
Potassium (mEq/L)	4.4 (4.0–4.8)

12-lead resting electrocardiography	
Atrial fibrillation	63 (27.2)
Implantable cardioverter-defibrillator	26 (11.2)
Pacemaker	124 (53.4)
Left bundle branch block	37 (15.9)
Right bundle branch block	93 (40.1)
Left anterior fascicular block	91 (39.2)
Low voltage of QRS	12 (5.2)
Ventricular premature contraction	108 (46.6)

2D-ecochardiography	
Left ventricular end-diastolic diameter (mm)	65 (59–71)
Left ventricular systolic diameter (mm)	55 (50–61)
Right ventricular diameter (mm)	25 (20–30)
Wall motion abnormalities	78 (33.6)
Left ventricular apical aneurysm	15 (6.5)
Left ventricular ejection fraction (%)	31.7 (24.5–40.0)

*N*: number of individuals; NYHA: New York Heart Association Functional Class.

**Table 2:** Association between chronic kidney disease or anemia and other baseline characteristics.

Baseline characteristics	CKD (*N* = 98)	Non-CKD (*N* = 134)	*P* value	Anemic (*N* = 41)	Nonanemic (*N* = 191)	*P* value
	Median (25^th^–75^th^) or *N* (%)			Median (25^th^–75^th^) or *N* (%)		
Clinical parameters						
Age (years)	63 (54–68)	52 (42–60)	<0.001	63 (52–70)	55 (44–64)	0.010
Gender (male)	59 (60.2)	94 (70.1)	0.114	32 (78.0)	121 (63.4)	0.720
NYHA classes I and II	61 (62.2)	96 (71.6)	0.171	25 (61.0)	132 (69.1)	0.323
NYHA classes III and IV	37 (37.8)	38 (28.4)		16 (39.0)	59 (30.9)	
Heart rate (beats/min)	66 (60–74)	70 (60–80)	0.026	70 (60–79)	68 (60–80)	0.873
Systolic blood pressure (mmHg)	100 (90–120)	110 (100–120)	0.150	110 (90–120)	110 (100–120)	0.640
Diastolic blood pressure (mmHg)	70 (60–70)	70 (60–80)	0.097	70 (60–80)	70 (60–80)	0.523
Type 2 diabetes mellitus	4 (4.1)	7 (5.2)	0.764	0 (0.0)	11 (5.8)	0.220

Laboratory analysis						
Sodium (mEq/L)	141 (137–144)	141 (138–144)	0.388	141 (135–145)	141 (138–144)	0.807
Potassium (mEq/L)	4.3 (4.0–4.7)	4.4 (4.1–4.8)	0.452	4.4 (4.1–4.9)	4.3 (4.0–4.8)	0.551

12-lead resting electrocardiography						
Atrial fibrillation	30 (30.3)	33 (24.6)	0.311	13 (31.7)	50 (26.2)	0.470
ICD	10 (10.2)	16 (11.9)	0.679	5 (12.2)	21 (11.0)	0.788
Pacemaker	65 (66.3)	59 (44.0)	0.001	27 (65.9)	97 (50.8)	0.079
Left bundle branch block	12 (12.2)	25 (18.7)	0.188	7 (17.1)	30 (15.7)	0.828
Right bundle branch block	37 (37.8)	56 (41.8)	0.536	21 (51.2)	72 (37.7)	0.109
Left anterior fascicular block	36 (36.7)	55 (41.0)	0.507	16 (39.0)	75 (39.3)	0.977
Low voltage of QRS	4 (4.1)	8 (6.0)	0.521	3 (7.3)	9 (4.7)	0.449
Ventricular premature contraction	41 (41.8)	67(50.0)	0.218	24 (58.5)	84 (44.0)	0.090

2D-ecochardiography						
Left ventricular end-diastolic diameter (mm)	66 (60–71)	65 (59–71)	0.497	64 (59–74)	65 (59–71)	0.856
Left ventricular systolic diameter (mm)	56 (50–62)	55 (50–61)	0.544	56 (47–64)	55 (50–61)	0.839
Right ventricular diameter (mm)	27 (22–31)	23 (18–30)	0.011	25 (20–31)	24 (20–30)	0.650
Wall motion abnormalities	32 (32.7)	46 (34.3)	0.790	10 (24.4)	68 (35.6)	0.168
Left ventricular apical aneurysm	5 (5.1)	10 (7.5)	0.470	2 (4.9)	13 (6.8)	1.000
Left ventricular ejection fraction (%)	32.0 (23.7–39.0)	31.7 (25.0–40.8)	0.462	31.1 (24.4–38.3)	31.7 (24.4–40.1)	0.590

CKD: chronic kidney disease; *N*: number of individuals; NYHA: New York Heart Association Functional Class; ICD: implantable cardioverter-defibrillator.

**Table 3:** Cox proportional hazard model analysis for independent predictors of all-cause mortality.

	Univariate			Multivariate		
All patients	HR	95% CI	*P* value	HR	95% CI	*P* value
Age (years)	1.00	0.99–1.01	0.876			
Gender (male)	1.27	0.88–1.84	0.207			
NYHA I functional class	0.54	0.37–0.77	0.001			
Heart rate (beats/min)	1.01	1.00–1.02	0.056			
SBP (mmHg)	0.98	0.97–1.00	0.006	0.99	0.98–1.00	0.015
DBP (mmHg)	0.98	0.96–1.00	0.015			
ICD	0.60	0.34–1.04	0.068	0.48	0.27–0.85	0.012
LAFB	1.63	1.16–2.28	0.005	1.52	1.08–2.13	0.017
LVDD (mm)	1.05	1.03–1.07	<0.001	1.04	1.02–1.06	<0.001
Serum sodium level (mEq/L)	0.93	0.89–0.96	<0.001	0.95	0.92–0.99	0.020
Anemia status	1.31	0.86–2.00	0.207			
CKD status	1.21	0.86–1.70	0.271			

HR: hazard ratio; CI: confidence interval; NYHA: New York Heart Association Functional Class; SBP: systolic blood pressure; DBP: diastolic blood pressure; ICD: implantable cardioverter-defibrillator; LAFB: left anterior fascicular block; LVDD: left ventricular end-diastolic diameter; CKD: chronic kidney disease.

## Data Availability

The datasets generated and/or analyzed during the current study are not publicly available due to the use of potentially identifying postal codes in the deprivation analysis, as approved by the local Human Research Ethics Committee, but they are available upon reasonable request.
